# The *Accordion* Zebrafish *tq206* Mutant in the Assessment of a Novel Pharmaceutical Approach to Brody Myopathy

**DOI:** 10.3390/ijms25179229

**Published:** 2024-08-25

**Authors:** Eylem Emek Akyürek, Francesca Greco, Chiara Tesoriero, Francesco Dalla Barba, Marcello Carotti, Giulia Gorni, Dorianna Sandonà, Andrea Vettori, Roberta Sacchetto

**Affiliations:** 1 Department of Comparative Biomedicine and Food Science, University of Padova, viale dell’Università 16, 35020 Legnaro, Italy; eylememek.akyurek@unipd.it; 2 Department of Biotechnology, University of Verona, strada Le Grazie 15, 37134 Verona, Italy; 3 Department of Biomedical Sciences, University of Padova, via U. Bassi 58/b, 35131 Padova, Italy

**Keywords:** skeletal muscle diseases, zebrafish, Sarco(endo)plasmic reticulum Ca^2+^-ATPase isoform 1 (SERCA1), human Brody disease

## Abstract

Brody disease (BD) is an “ultra-rare” human genetic disorder of skeletal muscle function due to defects in the *atp2a1* gene causing deficiency of the SERCA protein, isoform1. The main clinical signs are exercise-induced stiffness and delayed muscular relaxation after physical exercises, even mild ones. No mouse model nor specific therapies exist for Brody myopathy, which is therefore considered an orphan disease. Bovine congenital pseudomyotonia (PMT) is a muscular disorder characterized by an impairment of muscle relaxation and is the only mammalian model of human BD. The pathogenetic mechanism underlying bovine PMT has been recently clarified. These findings prompted us to purpose a potential pharmacological approach addressing a specific population of BD patients who exhibit reduced expression but still exhibit activity of the SERCA1 pump. Preclinical research involving in vivo studies is essential and necessary before clinical trials can be pursued and SERCA protein shows a high degree of conservation among species. So far, the only animal models available to study BD in vivo are a group of zebrafish mutant lines known as accordion zebrafish (acc). In this paper, we focused on a comprehensive characterization of the “acctq206” zebrafish variant. Our aim was to use this mutant line as an experimental animal model for testing the novel therapeutic approach for BD.

## 1. Introduction

Brody disease (BD) is an “ultra-rare” human genetic disorder of skeletal muscle function due to defects in the *atp2a1* gene [1]. Mutations in the *atp2a1* gene cause deficiency of the SERCA protein isoform1 (SERCA1). SERCA1 is exclusively expressed in fast-twitch (type 2) skeletal muscle fibers [2]. It is responsible for transporting Ca^2+^ from cytosol back into the lumen of sarcoplasmic reticulum (SR), playing a crucial role in skeletal muscle relaxation and in the maintenance of resting intracellular Ca^2+^ concentration. The main clinical signs of Brody myopathy are exercise-induced stiffness and delayed muscular relaxation after physical exercises, even mild ones. The muscles involved are predominantly voluntary muscles used for movement, such as the legs, arms, and eyelids. SERCA1 deficiency is responsible for impairment of muscle relaxation after contraction, due to prolonged increase of calcium concentration in skeletal muscle fiber cytoplasm [3]. At present, no mouse models [4] nor specific therapies [5,6] exist for Brody disease, which is therefore considered an orphan disease.

Bovine congenital pseudomyotonia (PMT), initially described in Chianina and subsequently in Romagnola breeds, is a muscular disorder characterized by an impairment of muscle relaxation. In PMT-effected animals, we identified, by DNA sequencing, missense mutations in the *atp2a1* gene. On the basis of clinical, genetic [7,8], and biochemical analyses [9], our group demonstrated that bovine congenital PMT represents an actual counterpart of Brody myopathy.

Using an in vitro experimental approach involving a heterologous cellular model overexpressing the mutant forms of SERCA1 found in Chianina and Romagnola cattle (i.e., R164H and G211V), the pathogenetic mechanism underlying bovine PMT [10,11] and consequently that of human BD has been recently clarified. It has been demonstrated that although the R164H or G211V mutations generate proteins which are corrupted during proper folding, which are ubiquitinated and prematurely degraded by the ubiquitin–proteasome system, SERCA1 mutants were catalytically active. In addition, proteasomal inhibition resulted in rescued SERCA1 mutants fully capable of maintaining cytoplasmic Ca^2+^ at similar levels to those in cells expressing the wild-type (WT) protein [10,11].

These findings prompted us to hypothesize that the retention of functionality in the mutated SERCA pump might be a fundamental requisite for the investigation of a potential pharmacological approach addressing the specific population of BD patients who exhibit reduced expression but preserved activity of the SERCA1 pump. This novel pharmacological approach is based on the repositioning strategy of “CFTR correctors” developed to treat type II mutations of cystic fibrosis transmembrane-conductance regulator (CFTR) (e.g., F508del) causing cystic fibrosis [12].

Preclinical research involving in vivo studies is essential and necessary before clinical trials can be pursued. SERCA protein shows a high degree of conservation among species. So far, the only animal models available to study BD in vivo are a group of zebrafish mutant lines known as accordion zebrafish (acc). Both Gleason et al. [13] and Hirata et al. [14] independently identified these mutants, which exhibit simultaneous contractions of trunk muscles on both sides of the embryo, leading to trunk shortening in response to touch. These phenotypic manifestations have been traced to mutations in the zebrafish SERCA1 gene, establishing a clear genetic and functional parallel to human BD.

The work of Gleason et al. and Hirata et al. [13,14] paved the way for using accordion mutants as animal models for studying the pathophysiology of BD. Among these lines displaying abnormal locomotion features, the acctq206 mutant carrying the missense S766F mutation remains the only one available today.

In this paper, we focused on a comprehensive characterization of the “acctq206” zebrafish variant. Our aim was to use this mutant as an experimental animal model for testing a novel therapeutic approach for BD. We focused specifically on the efficacy of correctors named C17 and C4, which have been shown in vitro to prevent the disposal of the R164H mutated SERCA1 (Patent W2014086687A1) and α-sarcoglycan (SG) mutants, causing bovine PMT and human limb–girdle muscular dystrophy (LGMDR3) [15], respectively.

## 2. Results

### 2.1. The S766F Missense Mutation in SERCA1 Induces Phenotypical Alterations and Development Delay in acctq206 Zebrafish Mutants

The acctq206 mutant line is characterized by a point mutation in the *atp2a1* gene [14] that replaces serine with phenylalanine at position 766 (S766F) of SERCA1 protein.

It has already been stated that homozygous Tq206^−/−^ die by ten days [14], whereas phenotypical analysis of heterozygous Tq206^+/−^ mutants has shown that they are vital, fertile, and able to produce viable offspring at the same time.

**Figure 1:**
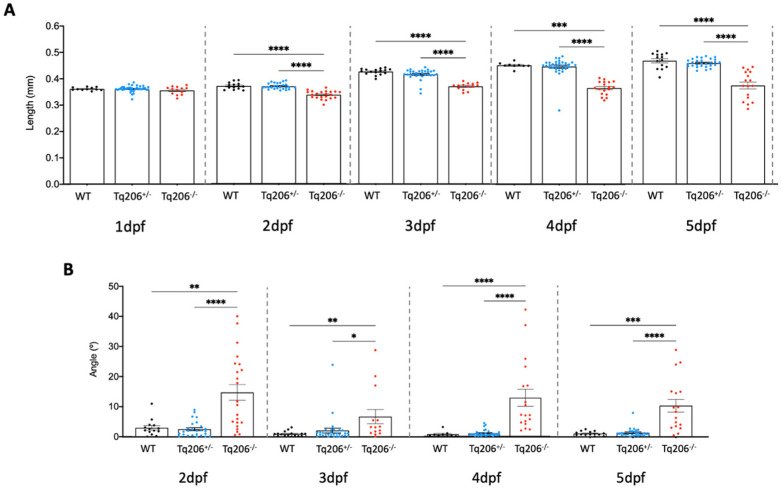
Morphometric assessment of Tq206^+/−^, Tq206^−/−^ and wild-type (WT) embryos/larvae. (**A**) Total body length differences between the experimental groups at 1, 2, 3, 4, and 5 days post-fertilization (dpf). (**B**) The tail curvature angle was measured from 2 dpf to 5 dpf of WT, Tq206^+/−^ and Tq206^−/−^ animals. Comparative analysis between groups was made with one-way analysis of variance (ANOVA). Nonparametric data were analyzed using the Kruskal–Wallis test, followed by Dunn’s post hoc test. Data are presented as mean ± SEM. *, *p* ≤ 0.05; **, *p* ≤ 0.01; ***, *p* ≤ 0.001; ****, *p* ≤ 0.0001.

From the in-cross of Tq206^+/−^ mutants, a total of 300 individuals were collected and subjected to morphological analysis from one to five days post-fertilization (dpf). This analysis encompassed the evaluation of three distinct parameters: total body length (Figure 1A), the angle of tail curvature (Figure 1B), and the presence or absence of the swim bladder (evaluated at 5 dpf) (Figure S4A,B). The morphological analysis of the population showed that from 48 h post fertilization (hpf), alterations in the total lengths of mutant embryos started to appear. In particular, Tq206^−/−^ embryos were shorter than WT embryos, while the heterozygous Tq206^+/−^ embryos did not present significant differences compared to WT (Figure 1A). After the examination of the tail curvature angle, Tq206^−/−^ siblings showed curved spine/tail compared to WT and Tq206^+/−^ siblings, confirming the presence of development alterations (Figure 1B).

At 5 dpf, the presence of the swim bladder was also considered as a part of morphological analysis. Interestingly, the proportion of Tq206^+/−^ larvae with swim bladder (75%) was quite similar to WT (71%), while in homozygous Tq206^−/−^ larvae, this structure was completely lacking (Figure S4B).

### 2.2. The S766F Missense Mutation in SERCA1 Impairs the Locomotor Activity of the acctq206 Zebrafish Larvae

The locomotor activity of both acctq206 mutants and their WT siblings was systematically assessed at 3 and 5 dpf. During each experimental session, larvae were subjected to a sequential alternation of 10-min periods of darkness followed by 10-min intervals of light stimulation.

**Figure 2:**
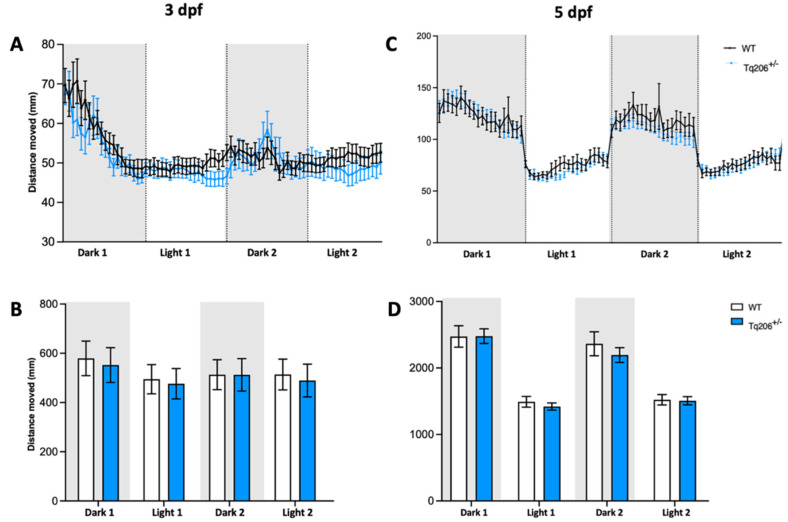
Behavioral assessment of Tq206^+/−^ and wild-type (WT) at different days post-fertilization (dpf). (**A**,**B**) Qualitative (**A**) and quantitative (**B**) analyses of the distance moved by 3-dpf-old Tq206^+/−^ (light blue line/bars) and WT (black line/white bars). Embryos underwent a period of 30 min of acclimatization followed by two alternating cycles of 10 min of darkness and 10 min of illumination, with the dark phases being indicated by grey boxes. (**C**) Distance moved by 5-dpf-old Tq206^+/−^ and WT embryos, and (**D**) their quantitative analyses. Data are presented as the mean ± SEM. Statistical analysis was conducted using a 2-way analysis of variance (ANOVA) with a Šidák’s multiple comparisons test.

At 3 and 5 dpf, qualitative observation of the total distance moved revealed that heterozygous Tq206^+/−^ siblings exhibited a behavior closely resembling that of the WT. Specifically, they showed a locomotor response characterized by an increase in distance traveled during the dark phases and a decrease in locomotor activity during the light phases, as depicted in Figure 2A,C. Quantitative analyses confirmed these observations (Figure 2B,D), and no statistically significant differences were observed between the Tq206^+/−^ mutants and their matched WT siblings.

**Figure 3:**
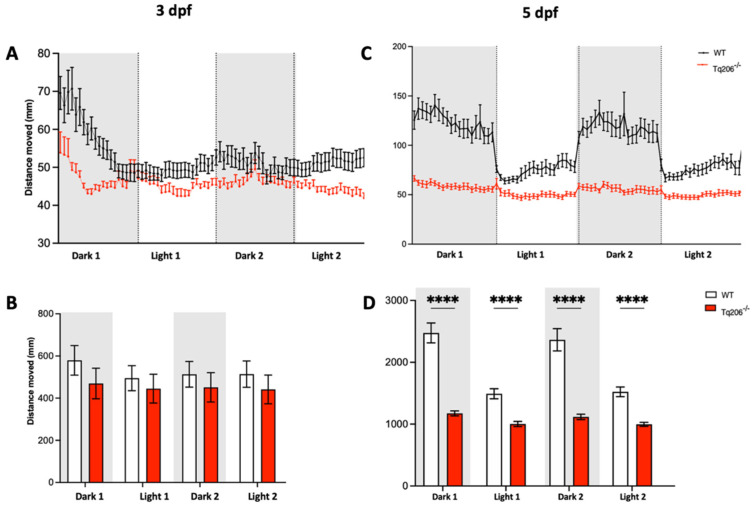
Behavioral assessment of Tq206^−/−^ and wild-type (WT) at different days post-fertilization (dpf). (**A**,**B**) Qualitative (**A**) and quantitative (**B**) analysis of the distance moved by 3-dpf-old Tq206^−/−^ (red line/bars) and WT (black line/white bars). Embryos underwent a period of 30 min of acclimatization followed by two alternating cycles of 10 min of darkness and 10 min of illumination, with the dark phases being indicated by grey boxes. (**C**) Distance moved by 5-dpf-old Tq206^−/−^ and WT embryos, and (**D**) their quantitative analyses. Data are presented as the mean ± SEM. Statistical analysis was conducted using a 2-way analysis of variance (ANOVA) with a Šidák’s multiple comparisons test. **** *p* < 0.0001.

Qualitative and quantitative observations, carried out on homozygous Tq206^−/−^ embryos at 3 dpf, showed an overlapping locomotor behavior compared to their WT siblings (Figure 3A,B). Interestingly, when the same locomotor analysis was performed in Tq206^−/−^ larvae at 5 dpf, we observed a statistically significant reduction of the distance traveled compared to the control WT sample (Figure 3C). Moreover, we noticed a total absence of response during the dark/light and light/dark transition, confirming the presence of a dramatic locomotor impairment associated with the depletion of the serca1 in zebrafish during development (Figure 3D).

### 2.3. Analyses of acctq206 Mutants Revealed the Presence of Altered Muscular Organization with Loss of Fiber Integrity

Via observation of zebrafish muscle under polarized light, muscle damage was detected as a reduction in birefringence, providing a sensitive indicator of muscle integrity [16].

**Figure 4:**
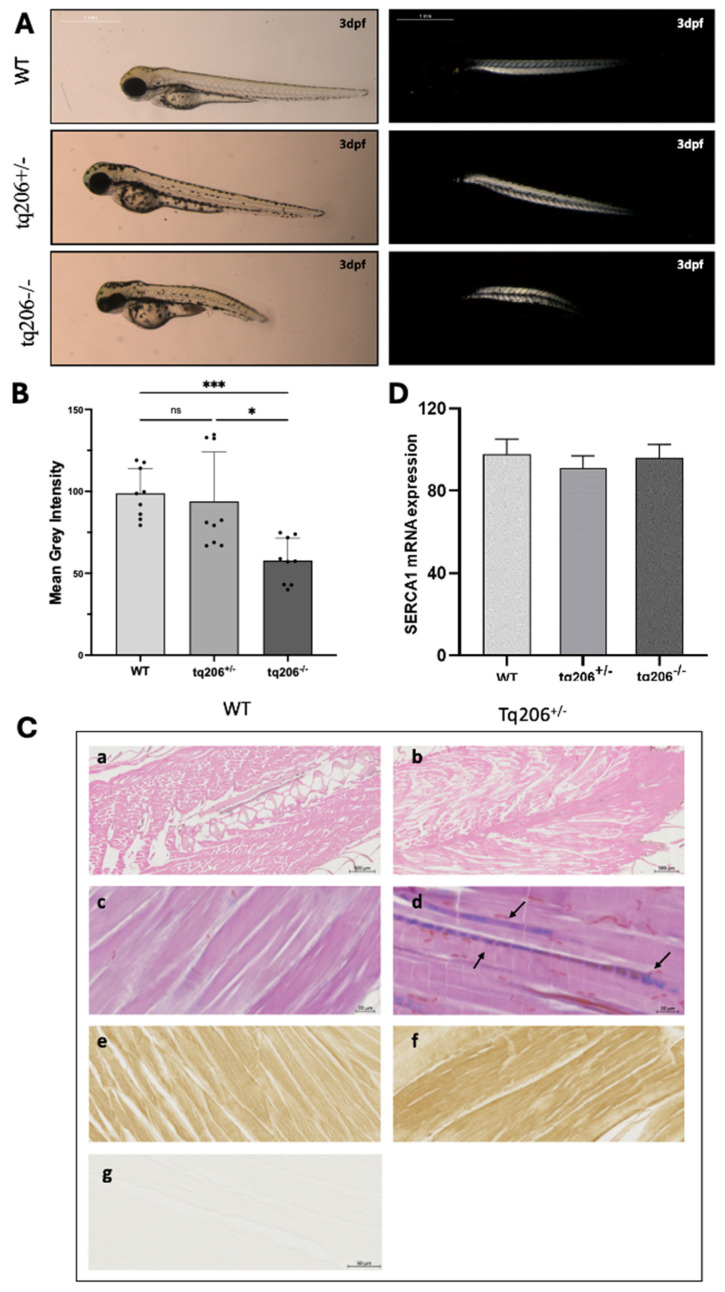
Muscle fiber integrity, the skeletal muscle histology and SERCA1 mRNA expression levels assessments. (**A**,**B**) Representative images (**A**) and quantification of birefringence (**B**) of wild-type (WT), Tq206^+/−^, and Tq206^−/−^ embryos are presented. Scale bar: 1 mm. Quantitative data are presented as mean ± SEM (Kruskal-Wallis test followed by Dunn’s post hoc test, ns, non-significant; *, *p* ≤ 0.05; *** *p* ≤ 0.001). (**C**) Hematoxylin & Eosin (panels (**a**,**b**), scale bar 500 µm), Azan-Mallory staining (panels (**c**,**d**), scale bar 50 µm), and SERCA1 immunostaining (panels (**e**,**f**), scale bar 20 µm) in muscle sagittal sections of WT (**a**,**c**,**e**,**g**) and Tq206^+/−^ (**b**,**d**,**f**) zebrafish. In panel d, arrows indicate fibrotic tissue (colored in blue) presence. Negative control of the immunohistochemistry is shown in (**g**). (**D**) SERCA1 mRNA expression levels in WT, Tq206^+/−^ and Tq206^−/−^, were quantified by Real Time RT-PCR. Data are presented as mean ± SEM.

As shown in Figure 4A, at 3 dpf, the sarcomeres of WT and heterozygous Tq206^+/−^ zebrafish exhibited a distinct and uniform brightness, with the signal uniformly distributed across the entire body. By contrast, at the same stage of development, Tq206^−/−^ mutant zebrafish embryos displayed markedly different birefringence patterns, showing a patchy birefringence signal indicative of muscle damage (Figure 4B) coupled with visible signs of tail curvature.

Further histopathological examinations (Figure 4C) were performed to determine whether the presence of the S766F mutation on SERCA1 protein might be associated with muscular alterations. Tissue sections from Tq206^+/−^ were stained using haematoxylin and eosin (H&E) (Figure 4C, panels a,b) and Azan-Mallory (A&M) (Figure 4C, panels c,d) protocols. Additionally, to investigate the effect of this mutation on the level of expression of SERCA1, immunohistochemical analysis was performed using specific anti-SERCA1 antibodies (Figure 4C, panels e,f). Results showed that the Tq206^+/−^ phenotype exhibited a less organized muscle structure characterized by the presence of fibrotic tissue interspersed within the muscular fibers (black arrow in Figure 4C, panel d). Furthermore, no significant difference in SERCA1 protein expression between Tq206^+/−^ and WT zebrafish was observed.

This observation was also confirmed by mRNA quantification made by quantitative real-time PCR (qPCR). With this analysis performed in 5dpf larvae, we demonstrated that the expression levels of Serca1 resulted identical in Tq206^+/−^, Tq206^−/−^ and WT control siblings (Figure 4D).

### 2.4. Treatment of acctq206 Mutants with the CFTR Molecule C17

**Figure 5:**
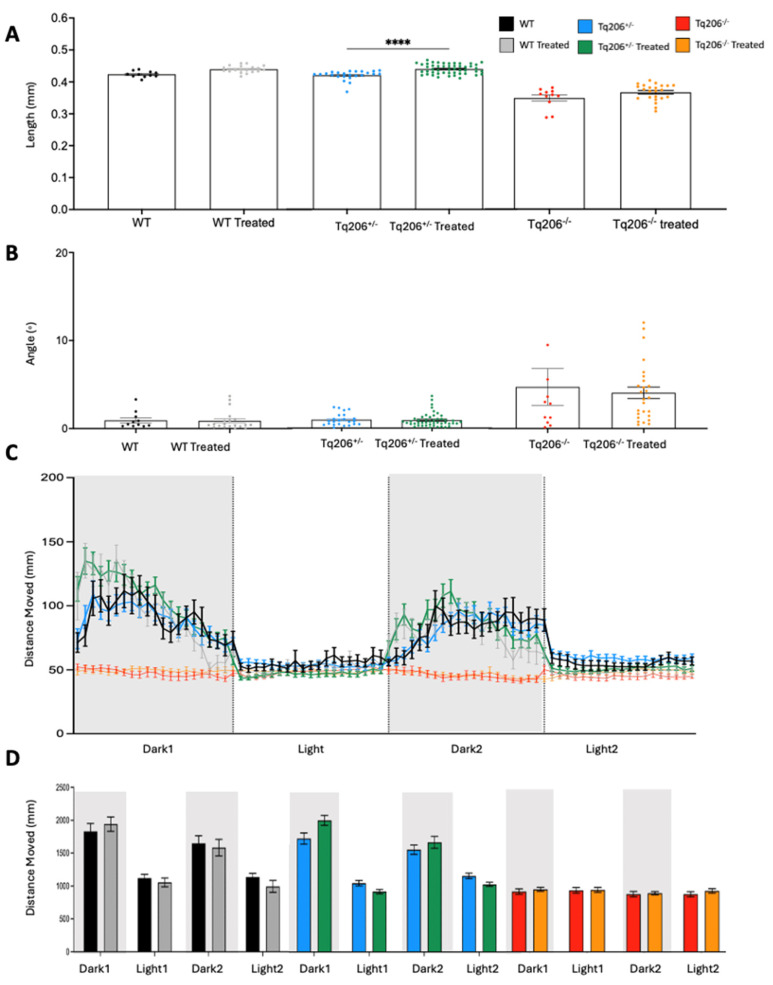
Morphometric (**A**,**B**) and behavioral assessment (**C**,**D**) of wild−type (WT), Tq206^+/−^, and Tq206^−/−^ zebrafish embryos after 48 h treatment with 0.05 μM CFTR corrector C17. (**A**) Total body length differences of treated and untreated WT, Tq206^+/−^ and Tq206^−/−^ zebrafish embryos. (**B**) Tail curvature angle measurements comparison with treated and untreated WT, Tq206^+/−^ and Tq206^−/−^ zebrafish embryos. Qualitative (**C**) and quantitative (**D**) analyses of the distance moved by treated and untreated WT, Tq206^+/−^ and Tq206^−/−^ zebrafish embryos. Embryos underwent 30 min of acclimatization followed by two alternating cycles of 10 min of darkness and 10 min of illumination, with the dark phases being indicated by grey boxes. All zebrafish embryos were 3 dpf old, and the treatment group received treatment of CFTR corrector C17 at 0.05 μM concentration for 48 h. Quantitative data are presented as mean ± SEM. Statistical analysis was performed by the Mann-Whitney test; **** *p* < 0.0001.

To assess the effects of C17 corrector in vivo, embryos obtained by the incross of Tq206^+/−^ zebrafish were treated for 48 h at a concentration of 0.05 μM in fish water. Results are shown in Figure 5, at 3 dpf, a total of 96 WT and mutant siblings were systematically recorded by a video tracking system (Noldus) for the locomotor analysis and then morphologically evaluated. Following the conclusion of these analyses, the DNA of each embryo was extracted to match the genotype with phenotypic parameters analyzed (total length, the angle of tail curvature, locomotor activity).

During each locomotor experimental session, larvae were subjected to a sequential alternation of 10-min periods of darkness followed by 10-min intervals of light stimulation. This dark-to-light cycle, comprising 10-min phases, was repeated twice.

No significant differences were observed between the WT and WT C17-treated groups (black and grey color respectively), and the C17 compound did not exert toxic effects at the concentration used. Specifically, no differences in body length (Figure 5A) and tail curvature (Figure 5B) were detected. Both qualitative (Figure 5C) and quantitative (Figure 5D) assessments of the total distance moved indicated that WT siblings treated with C17 exhibited a similar behavior compared to untreated WT fish.

Interestingly, while the Tq206^+/−^ C17-treated embryos (green color) exhibited no differences in tail curvature angles (Figure 5B), they showed an increased body length in comparison to their untreated (blue color) heterozygous siblings (Figure 5A). Furthermore, treated embryos seemed to improve locomotor performance during the periods of darkness, particularly in the first dark interval of the light–dark cycle. Unfortunately, the difference with the untreated was not statistically significant, as shown by data from quantitative analysis (Figure 5D).

Finally, the Tq206^−/−^ embryos treated with C17 (orange color) did not exhibit any significant differences after treatment, neither in terms of body length (Figure 5A) or tail curvature angles (Figure 5B). Additionally, the treated embryos displayed, a locomotor impairment that was comparable to their untreated (red color) Tq206^−/−^ siblings, as indicated by qualitative and quantitative analyses (Figure 5C,D).

### 2.5. Expression Levels of S766F Mutant SERCA1 Protein Remain Unchanged in HeLa Cells after Treatment with CFTR Correctors C17 and C4

To better explore the effects of the S766F amino acid substitution on the SERCA1 protein, in vitro analyses were performed. Mutation in *atp2a1* gene coding for SERCA1 was introduced by site-directed mutagenesis into the full-length cDNA encoding the adult isoform of fast-twitch rabbit SERCA1. The cDNAs encoding WT or S766F mutant SERCA1 were then used to transiently transfect HeLa cells. Cells expressing WT and S766F mutant SERCA1 were incubated for 24 h with C17 and with C4 CFTR correctors at varying concentrations. The C4 together with C17, was one of the most promising molecules in rescuing SG mutants, causing LGMDR3 as demonstrated by Carotti et al. [15].

**Figure 6:**
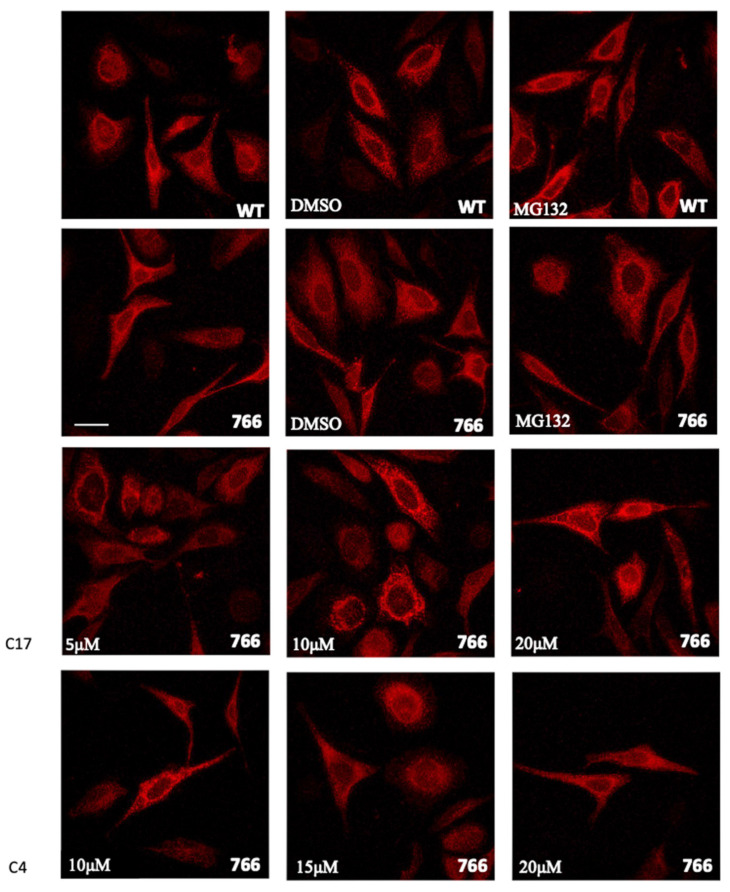
Cellular expression and localization of WT and mutant SERCA1 protein in HeLa cells. Cells were transfected with WT or with S766F mutated SERCA1 cDNAs, as indicated. Sixteen hours after transfection, MG132 (10 μM final concentration dissolved in DMSO), DMSO (its vehicle 0,1%) and different concentrations of C17 (5 μM, 10 μM, 20 μM) or C4 (10 μM, 15 μM, 20 μM) CFTR correctors, were added and cells were incubated for 8h. Transfected and treated cells were immunolabelled with monoclonal antibodies to SERCA1 and then incubated with the Alexa 568 red fluorescence secondary antibody. Images were recorded at the same setting conditions and magnification (scale bar 50 µm).

**Figure 7:**
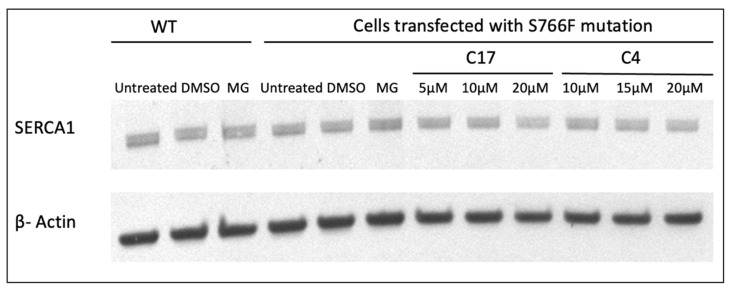
Representative Western blot showing the expression level analysis of WT, S766F mutant SERCA1 proteins after incubation with proteasome inhibitor MG132 and CFTR correctors at different concentrations. HeLa cells transfected with WT and S766F SERCA1 cDNAs were treated with the proteasome inhibitor MG132 (10 µM) or its vehicle DMSO (0.1%) and S766F mutants were also exposed to different concentrations of C17 and C4 CFTR correctors. An equal quantity of protein from total cell lysates was separated by SDS-PAGE and subjected to immunoblot analysis with antibodies specific to SERCA1 and 42 kDa beta-actin, used as loading control.

Immunostaining revealed in both WT and S766F mutant SERCA1 the typical staining pattern, i.e., a thin and diffuse network distributed throughout the cytoplasm (Figure 6) and that the expression level of WT and S766F variants was approximately equivalent. Furthermore, a nearly identical immunofluorescence pattern and signal were evidenced after the treatment with C17 or C4 CFTR correctors at all concentrations used or with proteasome inhibitor (MG132). These investigations were parallel to immunoblot analysis performed on total HeLa cell lysate using the identical specific antibody targeting SERCA1 (Figure 7). Results confirmed that the treatment with the two CFTR correctors or with MG132, did not induce an increase of the SERCA1 mutant content.

## 3. Discussion

BD is an “ultra-rare” muscular disorder without any therapeutic solution. At present, a mouse model is not available for Brody myopathy, as well as for many other muscular rare diseases.

We have designed and proven in vitro (Patent W2014086687A1 and paper in preparation), a novel specific pharmacological approach to this myopathy. This pharmacological treatment is based on the employment of molecules named CFTR correctors, developed to treat type II mutations of CFTR) (e.g., F508del) causing Cystic Fibrosis.

Bovine PMT represents to date the unique mammalian model of Brody myopathy but it is undoubtedly an unconventional model [7,17]. This reason prompted us to evaluate accordion zebrafish (acc) for its potential utility to assess the efficacy in vivo of the pharmacological approach proposed. These zebrafish mutant lines were identified independently and in the same year by Gleason et al. [13] and Hirata et al. [14]. They were named accordion zebrafish (acc) in analogy to the action of the musical instrument since mutants fail to coil their tails normally but contract the bilateral trunk muscles simultaneously to shorten the trunk just like the musical instrument. The excessive and prolonged contractions on both sides of the body interfere with the acquisition of patterned swimming responses. Further investigations showed that, as in Brody myopathy and in bovine PMT, acc mutants carry various defects in the *atp2a1* gene encoding SERCA1. Due to the similarities between the muscle phenotype and genetic defect of acc line and Brody myopathy, Hirata et al. [14] and Gleason et al. [13] indicated the zebrafish acc as an attractive animal model to study the human disease. Among acc lines described by these Authors in 2004, the mutant line named acctq206 is now commercially available.

With the aim of validating in vivo the efficacy of CFTR correctors, the main objective of this paper was the characterization and treatment of acctq206 mutant line with the novel therapeutic approach designed for BD. Specifically, we focused on the C17 molecule proven capable of successfully rerouting the human R98H-α-SG mutant to the sarcolemma of hind-limb muscles of a novel limb–girdle muscular dystrophy type 2D/R3 (LGMD2D/R3) in vivo in a murine model [18]. BD and LGMD, although different in symptoms and outcome, share with CF the same pathogenetic mechanism when mutations resulting in a folding defective protein are present.

Using the mutagenesis method, several mutations affecting SERCA1 expression/stability or its catalytic activity have been experimentally created in the past [19]. It was observed that some SERCA1 mutants, although stably integrated into the membrane, were expressed at low levels in heterologous cells [20]. It was therefore assumed that these defective pumps were recognized by a mechanism of detection and degradation of enzymes structurally defective and were degraded through proteolysis. On the other hand, it was also observed that SERCA1 pumps in which point-mutations affect the enzyme function, can escape surveillance mechanism and be normally expressed in cells, even though their catalytic function is compromised.

The retention of catalytical function in the mutant misfolded SERCA1 pump represents a fundamental requisite for the efficient exploitation of the potential innovative pharmacological approach here proposed (Patent W2014086687A1). On the basis of this premise, this potential therapeutic intervention is specifically addressed to that specific population of BD patients in which a reduced expression but activity of SERCA1 pump has been documented.

The zebrafish animal model offers several advantages, such as its small size, rapid development, external fertilization, and the abundant production of permeable and transparent eggs, which enable the study of pharmacological and toxicological responses, as well as the facilitation of morphological and behavioral analyses in both larvae and adult fish.

The characterization of the acctq206 was performed focusing mainly on phenotypical and behavioral analyses during embryogenesis and early developmental stages. Among morphological traits, the measurement of fish length was considered in previous works a good parameter to evaluate the presence of peculiar differences occurring in zebrafish mutant lines [21]. In this work, the angle of the dorsal curvature was measured as an additional parameter. Our data showed that heterozygous Tq206^+/−^ larvae had almost the same length as WT and did not present a dorsal curvature of the tail. Tq206^−/−^ siblings showed a more pronounced curved tail other than a lower length compared to WT or heterozygotes. Homozygotes Tq206^−/−^ pronounced curved tail larvae showed also a curved spine, representing the first sign of the myopathic phenotype in zebrafish. Moreover, Tq206^−/−^ larvae at 5dpf were also characterized by the total absence of swim bladder, which can be explained as an alteration in the development of mutant subjects.

In previous works [13,14], the locomotor behavior of fish was analyzed only for a limited period. To deeply investigate differences in muscle functionality, in this work, acctq206 mutants were monitored by a video tracking system able to record the fish motility for a prolonged time frame, during which different types of stimuli could be administrated. Larval swimming performance was evaluated via a light startle test in which zebrafish larvae were subjected to cycles of rapid changes in light intensity (i.e., dark:light cycle) where the sudden reduction in light was perceived by animals as a danger signal inducing a rapid swimming increase. While Tq206^+/−^ larvae and WT siblings exhibited similar locomotor activity at both 3 dpf and 5 dpf, Tq206^−/−^ siblings showed an overall lower locomotor activity. In addition, at 5 dpf, absence of response during the dark/light (and vice versa) transition was detected. 

Muscle integrity, assessed using birefringence imaging, indicated early (3 dpf) muscle damage in Tq206^−/−^ embryos. Altogether, these data confirmed that the S766F mutation in SERCA1 causes muscular impairment characterized by simultaneous bilateral contraction of trunk muscles that shorten the trunk length, as already described [13,14], resulting in impaired motility, resembling human Brody condition.

Administration of the CFTR C17 corrector to Tq206^−/−^ siblings exhibited no difference or improvement in morphological traits and swimming performance. In parallel, also Tq206^+/−^ C17 treated siblings with the exception of body length which was increased, showed no differences in terms of tail curvature angle and locomotor activity. These data seem to indicate that the C17 treatment did not ameliorate the physical or behavioral homozygous or heterozygous phenotypes associated with the S766F mutation, but at the same time, although preliminary, they suggest that the C17 molecule has no toxic effects at the concentration used in these experiments.

The mutation S766F on *atp2a1* did not affect mRNA transcription or stability, being the expression of the messenger at 5 dpf, the same as in Tq206^+/−^, Tq206^−/−^, and in WT. Nevertheless, immunoblot analysis of SERCA1 showed that the band intensity was too low in zebrafish embryos at this stage of development. Histopathologic and immunohistochemical staining performed on adult Tq206^+/−^ sibling evidenced a less organized muscle structure and the presence of fibrotic tissue but the absence of reduction in SERCA1 immunoreactivity. Altogether, these results suggested that the catalytic activity of SERCA1 is probably abolished by the mutation.

These conclusions were strengthened by the introduction of S766F SERCA1 substitution in the full-length cDNA encoding SERCA1 cloned in pcDNA3.1 expression vector and transfection in HeLa cells. Results confirmed that S766F SERCA1 mutant was quantitatively comparable with WT and that the treatment with C17 and C4 correctors or proteasome inhibitors MG132, did not affect the expression levels of mutated SERCA1.

SERCA1 pump comprises a large cytoplasmic headpiece, short luminal loops and a transmembrane domain made of 10 alpha-helices, named M1–M10 [22]. Alpha-helix M5 is depicted as the “spine” of the molecule and is highly conserved in mammals as well as in zebrafish. The mutation S766F is located in the portion of M5 domain called the “flexible hinge” (encompassing residues Ile765 to Asn768). Using the crystal structure of rabbit SERCA1, it has been shown that during the protein conformational change, occurring from the Ca^2+^-binding to the dissociation state, the middle part of the M5 domain rotates approximately 30°. This result is consistent with the functional role of the flexible hinge. In studies of SERCA1 mutagenesis, it has been evidenced that most mutations introduced in M5 disrupt or severely reduce SERCA1 function in vitro, enforcing the essential role of the M5 domain [23,24,25]. As previously stated by Gleason et al. [13] and Hirata et al. [14], the substitution of the polar side chain of Ser with the non-polar side chain of Phe consisting of a large aromatic benzene ring, at position 766 of M5 hinge region, is expected to restrict conformational freedom of the protein. This event could be the cause of disruption or severe reduction of SERCA1 function, without affecting the protein expression level and could explain at the same time, the extremely severe phenotype found in acctq206. This finding is in contrast to what described in bovine PMT. The replacement of the Arg side chain with the imidazole side chain of His or the substitution of the single neutral hydrogen atom of Gly with the hydrophobic side chain of Val in Chianina and Romagnola breed respectively, would destabilize SERCA1 structure, enhancing the susceptibility of the protein to degradation without inhibiting Ca2-ATPase activity, as previously demonstrated [10,11].

## 4. Materials and Methods

### 4.1. Animals

The study was conducted on WT and acctq206 mutant zebrafish lines, commercially available at the European Zebrafish Resource Center (Karlsruhe Institute of Technology). The stable mutant zebrafish model is characterized by a missense mutation inducing the substitution of cytosine at position 2297 on exon 17 with thymine (C −> T) in the *atp2a1* gene [14]. The mutation was verified by DNA sequencing using the Sanger method in conjunction with PCR (Figure S1). As additional genotyping methods, the presence of a specific restriction site at position 8160 of the *atp2a1* gene was investigated. The cleavage at this site by the restriction enzyme BfmI (SfcI), yields DNA fragments of varying lengths depending on the genotype of the examined samples (Figure S2). 

### 4.2. Ethics Statement

All husbandry and experimental procedures were carried out in accordance with the recommendations of the European Legislation for the Protection of Animals used for Scientific Purposes (Directive 2010/63/EU) [26] and of the Italian Parliament (L.LGS n26/2014). The project was examined and approved by the Ethics Committee of the University of Padua with protocol number 02/2022 (D2784.N.FRB).

### 4.3. Zebrafish Maintenance

Zebrafish were maintained in the facility of the University of Padova according to the standard protocols described by Kimmel et al. [27]. Animals were kept at 28 °C in conditioned saline aerated aquaria system under 12 h of light and 12 of dark. For mating, males and females were separated in the late afternoon and were freed the next morning to start courtship, which ended with egg deposition and fecundation. Oocytes were maintained at 28 °C in fish water (0.5 mM NaH_2_PO_4_, 0.5 mM NaHPO_4_, 0.2 mg/L methylene blue, 3 mg/L instant ocean pH 7–8) for development.

### 4.4. Genotyping of acctq206 Mutant Zebrafish

Following anaesthetization with tricaine methane sulfonate (0.16 mg/mL), zebrafish samples, either adult caudal fins or larvae, were lysed in 50 mM NaOH (Sigma-Aldrich, St. Louis, MO, USA) solution and incubated at 95 °C for 20 min. The lysates were neutralized by adding 1/10 of the sample volume of 1 M Tris HCl pH 8 (Sigma-Aldrich, St. Louis, MO, USA) and used as template for PCR. Genomic fragments of the targeted regions were amplified using standard PCR cycling conditions (Taq polymerase from ThermoFischer Scientific, Waltham, MA, USA) with specific primers (see Table S1). Amplicons were then incubated with the restriction enzyme BmfI (sfcI) (ThermoFischer Scientific, Waltham, MA, USA) according to the manufacturer’s instructions. The DNA fragments restriction pattern was read by electrophoresis and visualized by ChemiDoc Gel Imaging System (Bio-Rad, Hercules, CA, USA). Genotypes were confirmed by Sanger Sequencing performed by the Eurofins Genomics company (Anzinger Str. 7A, 85560 Ebersberg, Germania) and BMR Genomics Sequencing facility (BMR Genomics, Padova, Italy).

### 4.5. Morphological Analyses

A total of 300 larvae were used for the morphological phenotyping. Following anesthetization by tricaine methanesulfonate (160 mg/L), larvae at 1, 2, 3, 4 and 5 dpf (days post fertilization) were photographed using a Leica M205 FA stereomicroscope (Leica Microsystems, Heerbrugge, Switzerland). Measurements were performed offline by using the ImageJ v.153K software package Fiji. In particular, the total length (distance between the anterior tip of the head and the base of the caudal fin, as shown Figure S3A,B) was measured at all the time points of interest while the angle of the dorsal curvature was evaluated starting from 2 dpf onwards (Figure S3C). Moreover, the presence/absence of the swim bladder was also evaluated at 5 dpf (Figure S4A). At the end of the procedure, larvae were sacrificed and processed for the genotyping procedure.

### 4.6. Behavioral Analysis

Behavioral analysis was carried out by the DanioVision system and EthoVision XT software (Noldus Information Technology, Wageningen, The Netherlands), which allows for a high throughput tracking of zebrafish larvae. Analyses were performed at 3 and 5 dpf on zebrafish mutants and dpf matched WT. Larvae were individually placed in 48 multiwell plates with a constant temperature of 28 °C and a constant final volume of 0.5 mL of fish water. Behavioral experiments were recorded at ZT 1–6 (Zeitgeber time, ZT, 0 corresponds to the lights-on time). Larval swimming performance was evaluated under stressful conditions. Following 30 min of habituation under light conditions, larvae were consecutively exposed to 10 min of dark and 10 min of light stimulus. This dark:light cycle of 10:10 min was repeated two times. The sudden reduction in light is perceived by zebrafish as a danger signal inducing a rapid swimming increase.

### 4.7. Birefringence Assay and Densitometry of the Birefringence Pictures

Anaesthetized larvae at 3 dpf were placed in 2% methylcellulose and were analyzed between two glass polarizing filters under a Leica M80 stereomicroscope (Leica Microsystems, Heerbrugge, Switzerland). Larvae were photographed with a Leica MC170HD digital camera (Leica Microsystems, Heerbrugge, Switzerland). Pixel intensity in the trunk region was measured by the ImageJ v.153K software. The average grey value of the pixels in this area was measured, as a result, the mean grey value was considered.

### 4.8. Histological Analysis and Immunohistochemistry

Adult zebrafish were anaesthetized, fixed in 4% paraformaldehyde solution for 48 h, and subsequently decalcified in EDTA solution for 7 days. Dehydration, paraffin embedding, sectioning (5 µm of thickness) and staining with Azan-Mallory and haematoxylin-eosin were done according to standard protocols. Images were acquired by an automated slide scanner (Axioscan 7, Zeiss, Germany).

Immunostaining was carried out on fixed zebrafish tissue sections with rabbit-polyclonal antibodies against SERCA1 (dilution 1:1000, Cell Signaling Technology, Danvers, MA, USA) followed by biotinylated horse anti-mouse IgG antibody (2 µg/mL dilution; Vector Laboratories Inc., BA-2000). Reactions were done using a VECTASTAIN^®^ ABC Kit Peroxidase together with the Vector^®^ Diaminobenzidine (DAB) Kit (Vector Laboratories Inc., Ingold Road, Burlingame, CA, USA).

### 4.9. Real-Time Reverse-Transcript Polymerase Chain Reaction (RT-PCR)

Total RNA extraction and cDNA synthesis were performed as previously described [11]. All the samples were made of at least 30 pooled 5 dpf-old Tq206^+/−^, Tq206^−/−^ or WT larvae. Real-time PCR reaction conditions were as follows: 10 min at 95 °C, followed by 40 cycles of 15 s at 95 °C and 1 min at 60 °C. The specific primers used are listed in Table S1.

### 4.10. Evaluation of the Corrector C17 Effects on acctq206 Mutants

The C17 molecule was purchased from Rosstek Limited (Paralimni, Cyprus). The 0.05 μM of C17 treatment was conducted for 48 h on embryos obtained by the incross of Tq206^+/−^ acctq206 zebrafish. At 3dpf a total number of 96 WT and mutant siblings were morphological evaluated. At the end of the analysis, the DNA of each embryo was extracted for genotyping.

### 4.11. Morphological and Behavioral Analyses after Corrector C17 Treatment

For the C17 treatment, 1 dpf-old acctq206 embryos were dechorionated and placed in a Petri dish with fish water. C17 (0.05 μM) was added to the medium from 1 to 3 dpf with a final volume of 30 mL, and then washed before the locomotion test. In this experiment, each recording session lasted 1 h and 10 min and untreated acctq206 were used as controls. The results were analyzed at 3dpf as described before. 

### 4.12. SERCA1 Construct and Site-Directed Mutagenesis

The full-length adult rabbit SERCA1 cDNA was cloned in pcDNA3.1 expression vector as previously described [10]. The S766F substitutions were generated by using the QuikChange site-directed mutagenesis kit (Stratagene, San Diego, CA, USA), according to the manufacturer’s specifications. Primers used for the mutagenesis are shown in Table S1. The construct was verified by sequencing.

### 4.13. Cell Culture, Transfection and Treatment with CFTR Correctors and Proteasome Inhibitor

HeLa cells (ATCC, Manassas, VA, USA) were counted, seeded and grown in DMEM high glucose medium supplemented with 10% FBS. Cells were transfected with WT and S766F SERCA1 mutant cDNA, using jetOPTIMUS^®^ DNA (Polyplus Transfection, New York, NY, USA) transfection reagents, according to the manufacturer’s instructions. Sixteen hours after transfection, cells were incubated for 8 h with C17, C4 CFTR correctors and with MG132 (10 μM final concentration dissolved in DMSO) (Sigma-Aldrich, St. Louis, MO, USA). After treatment, cells were lysed as described [11]. Protein concentrations were determined by the Bicinchoninic Protein Assay Kit (Quantum Protein Assay Kit, EuroClone, Pero, MI, Italy).

### 4.14. Gel Electrophoresis and Immunoblotting

Proteins were resolved by sodium dodecyl sulfate–polyacrylamide gel electrophoresis (SDS-PAGE) and transferred to a nitrocellulose membrane. Membranes were probed with antibodies against SERCA1 (dilution 1:5000, ThermoFisher Scientific, Waltham, MA, USA) or β-actin (dilution 1:30,000, Sigma Aldrich, St. Louis, MO, USA) as described in Akyürek et al. [11] and developed with BCIP/NBT solution or ECL chemiluminescent substrate, respectively. The blots were imaged with iBright 1500 (Thermo Fischer Scientific, Waltham, MA, USA). 

### 4.15. Immunofluorescence Analysis

HeLa cells grown on 13 mm glass coverslips were transfected with WT and S766F SERCA1 mutant cDNA. After transfection and treatment with C17, C4 CFTR correctors and with MG132 (10 μM final concentration dissolved in DMSO) cells were fixed with 4% paraformaldehyde and immunodecorated with primary antibodies, mouse monoclonal against SERCA1 (dilution 1:500, ThermoFisher Scientific, Waltham, MA, USA) and subsequently with Alexa Fluor 568 red secondary antibody (dilution 2 μg/mL, ThermoFisher Scientific, Waltham, MA, USA) as described [11]. Glass coverslips were closed with mowiol (Sigma-Aldrich, St. Louis, MO, USA). Confocal microscopy was performed using a TCS-SP5 II confocal laser scanning microscope (Wetzlar, Germany).

### 4.16. Statistics

Statistical analysis was made with GraphPad Prism 10 software. The Kruskal–Wallis test and 2-way ANOVA with the Sidak post hoc test and Mann–Whitney test were used. A level of confidence of *p* < 0.05 was used for statistical significance.

## 5. Conclusions

As previously stated, the retention of functional properties of mutated SERCA pumps is essential for the efficacy of the potential innovative pharmacological therapy; based on CFTR correctors, we proposed to cure Brody patients. The mutation found in the zebrafish line acctq206 unfortunately did not meet this requisite, and, as a result, treatment with C17 had no beneficial effects in this zebrafish model. However, the current results indicate that C17 CFTR corrector is well tolerated in this animal model. For this reason, using of the Crispr Cas9 technique we planned to create two zebrafish mutant lines with the same mutations on the SERCA1 gene described in the bovine PMT.

## Data Availability

The data presented in this study are available in the article and in the Supplementary Materials.

## Supplementary Materials

The following supporting information can be downloaded at: https://www.mdpi.com/article/10.3390/ijms25179229/s1.
