# Supplementary material for: The Accordion Zebrafish tq206 Mutant in the Assessment of a Novel Pharmaceutical Approach to Brody Myopathy
Source: Int J Mol Sci. 2024 Aug 25;25(17):9229. doi: 10.3390/ijms25179229 (PMC11395142; doi:10.3390/ijms25179229)
Supplement: Supplementary file 1 [file ijms-25-09229-s001.zip › ijms-3105403-supplementary.pdf]

Supplementary material

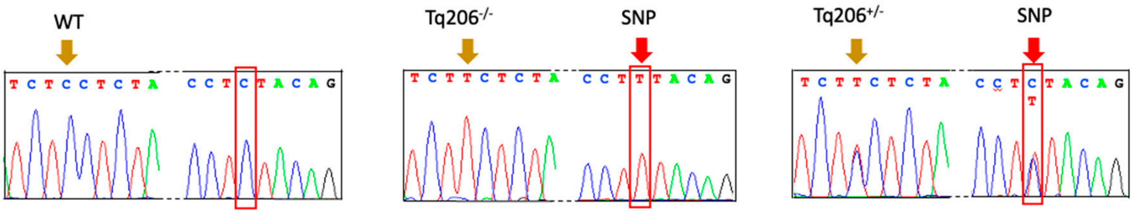

Figure S1 Representative Sanger sequencing chromatograms of WT, Tq206+/- and Tq206-/- zebrafish. Yellow arrows indicate the point mutation changing according to the genotype, while the red arrows represent the SNP.

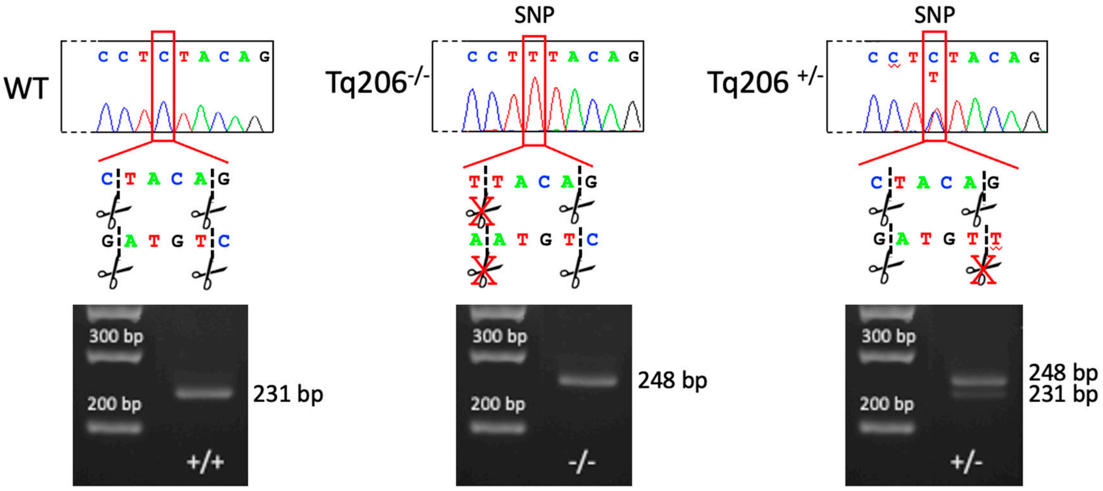

Figure S2 Representative agarose gel of the bands' pattern generated by PCR followed by BfmI restriction. WT and Tq206-/- animals were identified by one band at 231 and 248 base pairs (bp), respectively. In contrast, Tq206+/- were identified by the presence of two bands: one at 231 bp, one at 248 bp. A positive control (CP) of a 282 bp long DNA fragment was run in parallel.

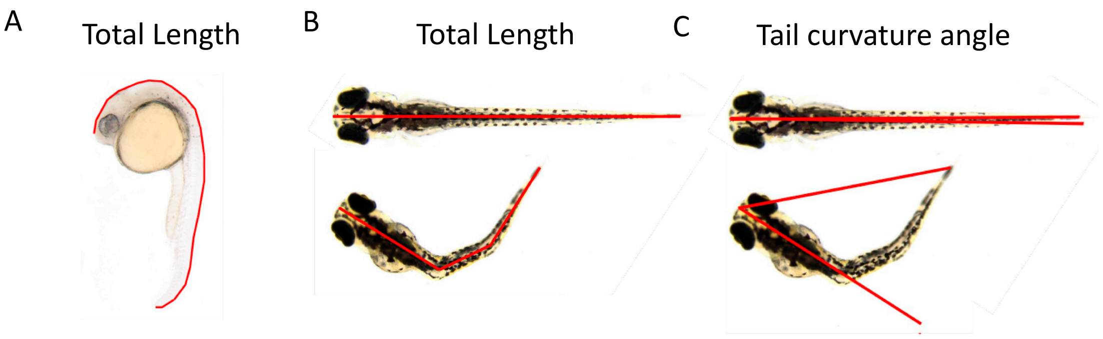

Figure S3 Representative body length measurements and the tail curvature angle in WT, Tq206+/- and Tq206-/- embryos/larvae. (A) Measurements method of the body length till the age of 2dpf. (B) Measurement method of body length after the age of 3dpf. (C) Measurement method of tail curvature angle.

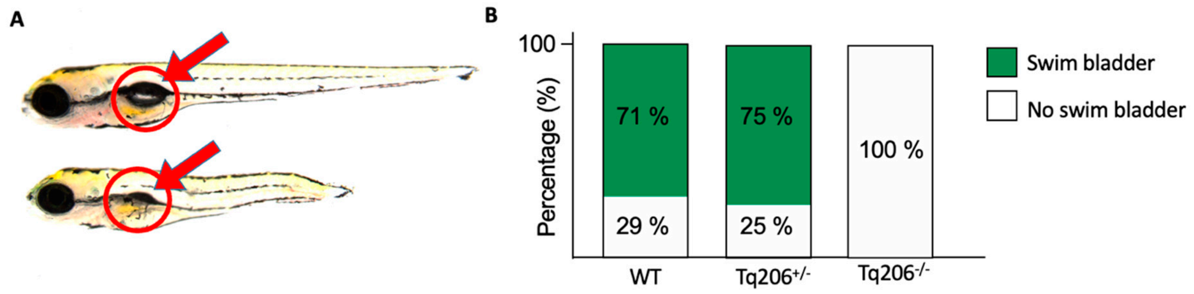

Figure S4 Evaluation of the presence/absence of the swim bladder at 5 days post-fertilization in WT, Tq206+/- and Tq206-/- embryos/larvae. (A) Representative images of the swimming bladder position in WT and Tq206-/- zebrafish. (B) The quantitative data are expressed in the form of percentages. Sample size: N_{WT}= 14, $N_{Tq206^{+/-}}$= 28, $N_{Tq206^{-/-}}$.= 17.

PCR + Sanger; forward	5’-GGCCGACGACAACTTCTCTA-3’
PCR + Sanger; reverse	5’-TGGGATCAGAGCCTCAGGAA-3’
PCR + Enzyme restriction; forward	5’-GGCCGACGACAACTTCTCTA-3’
PCR + Enzyme restriction; reverse	5’-TGGGATCAGAGCCTCAGGAA-3’
RT-PCR; SERCA1 forward	5'- CCT GAC TGC AGC TCT TGG TCT T -3'
RT-PCR; SERCA1 reverse	5'- AAC CTC CAA TGG CCA GGT ATC T -3'
RT-PCR EF1a forward	5'- ACA TCG CCT GCA AGT TTG CT -3'
RT-PCR EF1a reverse	5'- CAA CGG TCT GCC TCA TGT CA -3
Site-Directed Mutagenesis forward	5’-CCGCTACCTCATCTTCTCCAACGTGGGCG-3’
Site-Directed Mutagenesis reverse	5’-CGCCCACGTTGGAGAAGATGAGGTAGCGG-3’

Table S1. Primers for PCR, RT-PCR and Site-Directed Mutagenesis method.
